# Burrowing of urinary bladder wall by the tip of a size 22 Fr silicone foley catheter in an adult male patient with multiple sclerosis and suprapubic cystostomy: should caution be exercised in using a size 22 Fr silicone foley catheter for long-term drainage of neuropathic bladder?

**DOI:** 10.1186/1757-1626-1-25

**Published:** 2008-07-08

**Authors:** Subramanian Vaidyanathan, Peter L Hughes, Paul Mansour, Bakul M Soni

**Affiliations:** 1 Regional Spinal Injuries Centre, District General Hospital, Southport, PR8 6PN, UK; 2 Department of Radiology, District General Hospital, Southport, PR8 6PN, UK; 3 Department of Cellular Pathology, District General Hospital, Southport, PR8 6PN, UK

## Abstract

**Introduction:**

Silicone Foley catheters tend to become stiffer as size of the catheter increases. Whereas the tip of a size 12 French silicone, Foley catheter is soft and flexible, a size 24 French silicone, Foley catheter is distinctly stiff. Chronically inflamed neuropathic bladders are susceptible to perforation by the tip of a Foley catheter. We report a patient with multiple sclerosis and moderately severe chronic cystitis, in whom a size 22 French Foley catheter burrowed through the dome of urinary bladder.

**Case presentation:**

A 55-year-old, Caucasian male suffering from multiple sclerosis underwent suprapubic cystostomy in January 2007. Initially, a size 16 Fr. silicone, Foley, catheter was inserted. During subsequent catheter changes, silicone Foley catheters of progressively increasing sizes were inserted and in July 2007, a size 22 Fr. catheter was used in order to prevent blockages and consequent bypassing of urine. In April 2008, he had an uneventful change of suprapubic catheter; but a week later, this patient developed profuse bypassing. On examination, suprapubic catheter contained fresh blood; there was hardly any urine in the leg bag, which was attached to suprapubic catheter. Cystogram showed localised extravasation of contrast on the superior aspect of urinary bladder around the tip of Foley catheter, which protruded beyond the dome of urinary bladder. The size 22 Fr. catheter was removed and a size 20 Fr silicone, Foley, catheter was inserted ensuring that the tip of catheter pointed towards bladder neck. This patient received gentamicin intravenously and he was prescribed ciprofloxacin for five days. He did not develop temperature or other features of sepsis. Bypassing stopped completely.

**Conclusion:**

In this patient, bladder biopsy had shown moderately severe chronic inflammation and congestion. We learn from this case that we should have used a smaller size catheter, which has a softer texture and changed the catheter at shorter intervals rather than insert a larger bore catheter, and run the risk of perforation of neuropathic bladder by the tip of a stiff Foley catheter.

## Introduction

Rupture of the bladder in persons with multiple sclerosis can result from a combination of several factors. Outflow obstruction from neurogenic sphincter disturbance, chronic bladder infection, direct mechanical trauma from an indwelling catheter, and potentiation of infection by corticotrophin may all play a part. Harding and associates reported bladder rupture in two patients with multiple sclerosis and both patients had chronically inflamed bladders [1].

Perforation of the urinary bladder associated with long-term indwelling catheter is a rare and lethal iatrogenic disorder. Hughes and associates [2] reported an 80-year-old-white man, who had Foley catheter drainage of urinary bladder for more than five years; in this patient, the tip of Foley catheter eroded through the dome of the bladder. Pressure necrosis from contraction of urinary bladder on catheter tip is believed to be the pathogenesis for erosion of bladder by the tip of a Foley catheter [3]. Spees and associates [4] reported three unusual situations of bladder perforation in patients who had a history of long-term urinary bladder catheterization: one in which a fibroid uterus probably played a role in pressure necrosis of a bladder with an indwelling catheter, one in which carcinoma of the prostate and faulty catheter drainage was present, and one in which pelvic radiation therapy was followed by bladder perforation. Neuropathic bladder with chronically inflamed bladder wall is another situation where the tip of long-dwelling catheter can erode into the bladder wall. We report a patient with multiple sclerosis and moderate active chronic inflammation of vesical mucosa, in whom the tip of a size 22 French silicone, Foley catheter burrowed through bladder wall.

## Case Report

**Figure 1:**
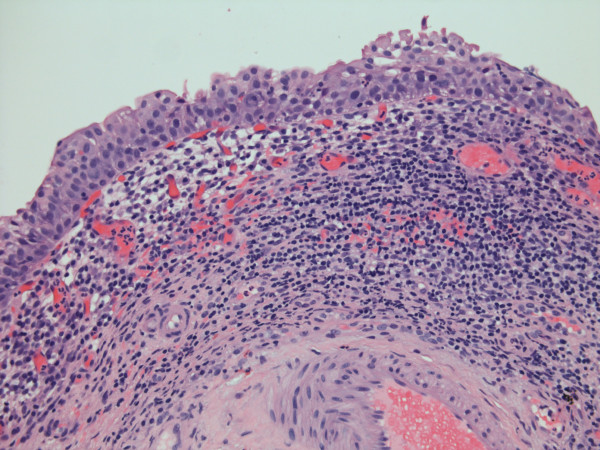
Microphotograph of bladder biopsy shows moderately severe chronic inflammation and congestion.

A Caucasian male, born in 1953, was diagnosed to be suffering from multiple sclerosis in 1992. He was doing intermittent catheterisation about four times a day and wore a penile sheath during night. But penile sheath would not stay and he would wet himself, which resulted in maceration of skin over penis, scrotum and buttocks. He was prescribed tolterodine, which made quite a significant improvement. However, in August 2002, he started using indwelling urethral catheter. He experienced no trouble with urine leakage but urethral catheter interfered with his sexual activity. Flexible cystoscopy showed a contracted bladder with reddened areas. In March 2003, biopsies were taken from these areas. Histology showed benign transitional epithelium and moderately severe chronic inflammation and congestion. (Figure 1)

In November 2006, he was found to have urethral split due to the indwelling urethral catheter. Therefore, suprapubic cystostomy was performed in January 2007. Initially, a size 16 Fr. Foley, silicone catheter was inserted for suprapubic drainage. In March 2007, the size of suprapubic catheter was increased to size 18 Fr. A size 20 Fr. silicone Foley catheter was inserted in May 2007, followed by a size 22 Fr. silicone Foley catheter in July 2007. Subsequently, suprapubic Foley catheter was changed after intervals of eight weeks, six weeks, four weeks, six weeks, six weeks, four weeks, six weeks, and then three weeks. He used to pass lot of sediments in urine and therefore, the catheter was changed at frequent intervals in order to prevent blockage of catheter and consequent bypassing of urine. He had very vulnerable skin over genitalia and any bypassing of urine could lead to maceration of skin over penis, scrotum and perineum.

The last catheter change was performed on 21 April 2008 when no difficulty was observed either during removal of old catheter or insertion of a new Foley catheter. There was no bleeding, and clear urine drained through suprapubic catheter while the patient was being observed in the hospital for 30 minutes after change of suprapubic cystostomy. A week later, the patient rang spinal unit to say that he was constantly wet, as most of times, he passed urine per urethra. Urine, which he drained through penis, was clear; there was no blood. He was advised to take modified-release oxybutynin 10 mg once daily on the assumption that he had bladder spasms. X-ray of urinary bladder was scheduled to look for vesical calculus, and cystogram was organised to check position of Foley catheter.

Four days later, the patient was examined prior to radiological studies. He was found to be wet from below. Suprapubic catheter contained fresh blood. There was hardly any urine in the leg bag, which was attached to suprapubic catheter. Following injection of 20 ml of Optiray 300 through suprapubic catheter, X-ray was taken. [Each millilitre of OPTIRAY 300 (ioversol injection 64%) contains 636 mg of ioversol with 3.6 mg of tromethamine as a buffer and 0.2 mg of edetate calcium disodium as a stabilizer. OPTIRAY 300 provides 30% (300 mg/mL) organically bound iodine.]

**Figure 2:**
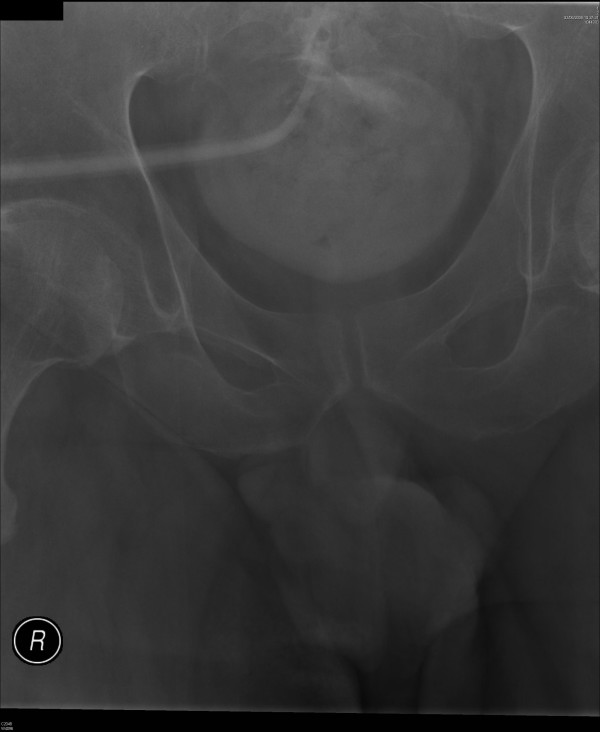
Following injection of 20 ml of Optiray 300 through suprapubic catheter, X-ray was taken. Cystogram showed that the tip of size 22 French Foley catheter had protruded beyond the dome of urinary bladder. There was suspicion of extravasation of small amount of contrast around the tip of Foley catheter.

**Figure 3:**
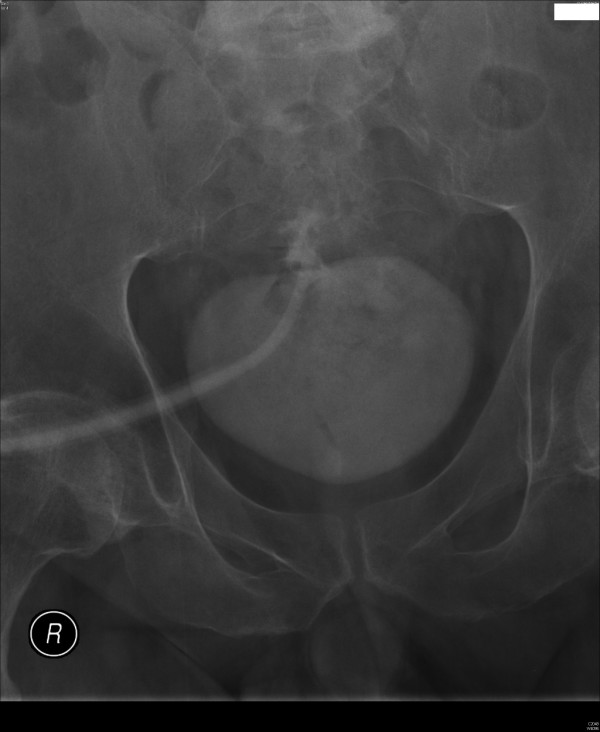
Another 20 ml of Optiray 300 was injected and X-ray was taken. This X-ray clearly demonstrated localised extravasation of contrast on the superior aspect of urinary bladder around the tip of Foley catheter.

Cystogram showed that the tip of Foley catheter had protruded beyond the dome of urinary bladder. (Figure 2) There was suspicion of extravasation of small amount of contrast around the tip of Foley catheter. Another 20 ml of Optiray 300 was injected and X-ray was taken. This X-ray clearly showed localised extravasation of contrast on the superior aspect of urinary bladder around the tip of Foley catheter. (Figure 3)

The size 22 French, silicone, Foley catheter was removed. A size 20 Fr. silicone, Foley catheter was inserted and it was ensured that the tip of Foley catheter pointed inferiorly towards the scrotum. This patient was given gentamicin 160 mg intravenously and was advised to take by mouth ciprofloxacin 500 mg twice a day for five days. He did not develop temperature. The size 20 French, Foley catheter continued to drain urine satisfactorily; he experienced no further bypassing.

## Discussion

Silicone Foley catheters tend to become stiffer as size of the catheter increases. Whereas the tip of a size 12 Fr. silicone, Foley catheter is very soft and flexible, a size 24 Fr. silicone, Foley catheter is distinctly stiff. In our enthusiasm to prevent blockages and consequent bypassing of urine, we used a size 22 Fr. Foley, silicone, catheter, which burrowed through the dome of bladder.

The spectrum of inflammatory changes seen in the mucosa of neuropathic bladder appears to be related to the presence or absence of long-term indwelling catheters. The degree and extent of inflammatory changes in the bladder mucosa of patients with spinal cord injury, who have been practising intermittent catheterisation, are markedly less, as compared to the biopsies taken from the neuropathic bladder of spinal cord injury patients with long-term indwelling catheters [5]. As neuropathic bladders in persons with long-term indwelling catheter show moderate to severe, acute and chronic inflammatory changes in vesical mucosa, it is likely that spinal cord injury patients or those with multiple sclerosis, who require long-term indwelling catheter drainage, are probably more susceptible to develop erosion of urinary bladder by the tip of a stiff Foley catheter than able-bodied individuals with healthy bladder, who generally require indwelling catheters only for a short duration.

In hindsight, we realise that we should have been prudent. We should have used a smaller size Foley catheter and changed the catheter at shorter intervals rather than inserting a larger bore catheter and running the risk of perforation of neuropathic bladder by the tip of a stiff Foley catheter.

## Conclusion

As neuropathic bladders in persons with long-term indwelling catheter show moderate to severe, acute and chronic inflammatory changes in vesical mucosa, it is likely that spinal cord injury patients or those with multiple sclerosis, who require long-term indwelling catheter drainage, are at risk of developing perforation of urinary bladder by the tip of a stiff Foley catheter. In hindsight, we learn that we should have used a smaller size catheter, which has a softer texture and changed the catheter at shorter intervals rather than insert a larger bore catheter, which is stiff, and run the risk of erosion of neuropathic bladder by the tip of a stiff Foley catheter.

## Patient's perspective

It is more comfortable not to be wet in myself. After the last change of suprapubic catheter, the size 22, silicone catheter worked for a short period. But within a week, I was bypassing. After insertion of a size 20 French, catheter, I am lot more confident; it seems to drain better. I do not have bypassing.

## Consent

Written informed consent was obtained from the patient for publication of this case report and accompanying images. A copy of the written consent is available for review by the Editor-in-Chief of this journal.

## Competing interests

The authors declare that they have no competing interests.

## Authors' contributions

SV suspected problem with suprapubic catheter and performed cystography. PM interpreted bladder biopsy slides. All authors contributed to the manuscript and approved the final version.
